# Deep learning-based diagnosis of temporomandibular joint osteoarthritis using whole-body bone scans

**DOI:** 10.1016/j.isci.2025.114027

**Published:** 2025-11-11

**Authors:** Yeon-Hee Lee, Hee-Sung Kim, Seonggwang Jeon, Q-Schick Auh, Il Ki Hong, Sunju Choi, Fernando Guastaldi, Hyungsoon Im, Yung-Kyun Noh, Akhilanand Chaurasia

**Affiliations:** 1 Department of Orofacial Pain and Oral Medicine, Kyung Hee University Dental Hospital, KyungHee University Medical Center, Kyung Hee University School of Dentistry, #613 Hoegi–dong, Dongdaemun–gu, Seoul 02447, Korea; 2 Center for Systems Biology, Massachusetts General Hospital, 185 Cambridge Street, Boston, MA 02114, USA; 3 Department of Computer Science, Hanyang University, Seoul 04763, Korea; 4 Department of Nuclear Medicine, Kyung Hee University Medical Hospital, Kyung Hee University Medical School, #613 Hoegi–dong, Dongdaemun–gu, Seoul 02447, Korea; 5 School of Computational Sciences, Korea Institute for Advanced Study (KIAS), Seoul 02455, Korea; 6 Division of Oral and Maxillofacial Surgery, Department of Surgery, Massachusetts General Hospital, Harvard School of Dental Medicine, 50 Blossom Street, Thier 513A, Boston, MA 02114, USA; 7 Department of Oral Medicine and Radiology, King George’s Medical University, Lucknow, Uttar Pradesh, India

**Keywords:** Orthopedics, Bioinformatics

## Abstract

Temporomandibular joint osteoarthritis (TMJ-OA) is a degenerative condition that causes pain and functional limitation, yet its relationship with systemic osteoarthritis (OA) remains unclear. This study developed deep learning models to automatically diagnose TMJ-OA using bone scintigraphy (bone scans) and to evaluate systemic OA features as potential predictors. A dataset of 1,943 patients (3,886 TMJs) was analyzed with three convolutional neural network (CNN) approaches based on the VGG16 architecture. In head-and-neck imaging, the VGG16-Lite model achieved outstanding diagnostic accuracy (AUC >0.90) across age and sex subgroups, outperforming pretrained models. Whole-body scans excluding the head and neck provided only modest predictive value for TMJ-OA (AUC ∼0.65), suggesting limited utility of systemic features alone. These findings highlight the value of targeted bone scans with lightweight deep learning models for robust and efficient TMJ-OA detection, while also underscoring the need for further research into systemic associations.

## Highlights

• Deep learning (VGG16-Lite) enables accurate TMJ-OA diagnosis on bone scans (AUC >0.90)
• TMJ-focused imaging outperforms whole-body scans (AUC >0.90 vs. ∼0.65)
• Lightweight model achieves high accuracy with low computational cost
• AI-assisted diagnostics may improve accessibility for TMJ-OA in clinical practice

## Graphical abstract

**Figure:**
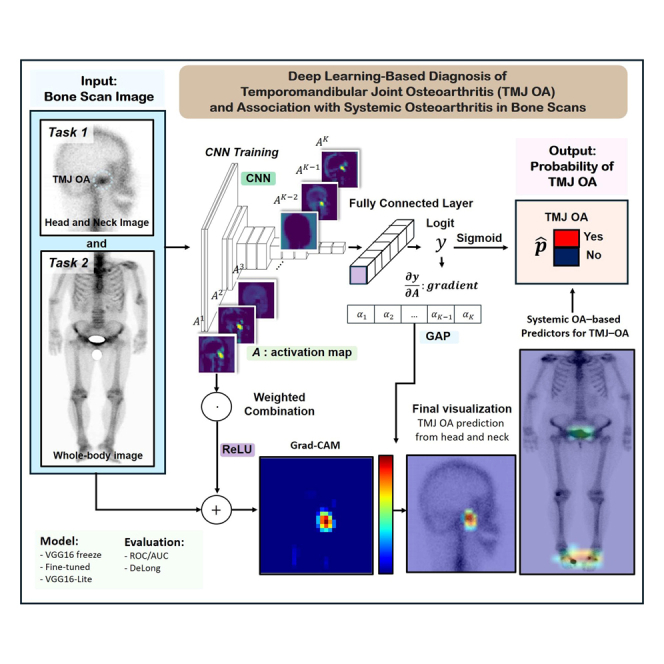

## Introduction

Temporomandibular joint (TMJ) osteoarthritis (OA) is a degenerative condition that represents a key subtype of temporomandibular disorder (TMD), affecting approximately 8%–16% of adults.^1^ It can arise from various causes, including excessive mechanical load, trauma, disc displacement, or developmental abnormalities.^2^ In patients with TMJ-OA, joint space narrowing and bony structural changes tend to worsen with age.^3^ Patients with TMJ-OA commonly experience joint pain, limited mandibular movement, and dysfunction, often accompanied by pathological changes in cartilage, subchondral bone, synovial lining, and adjacent soft tissues.^4^ This progressive nature of the disease often leads to a decline in patients’ quality of life.

Despite its clinical significance, the diagnosis of TMJ-OA remains challenging, particularly in its early or active stages. Cone-beam computed tomography (CBCT) and magnetic resonance imaging (MRI) are commonly employed in the evaluation of TMJ pathology, both modalities are primarily designed to detect structural alterations. CBCT provides detailed visualization of osseous components, such as subchondral sclerosis, cortical erosion, and osteophyte formation, while MRI is regarded as the gold standard for assessing soft tissue elements, including the articular disc and joint effusion.^5^^,^^6^^,^^7^ Nevertheless, these modalities often fail to detect subtle or early-stage degenerative changes that lack overt anatomical deformation. In contrast, bone scintigraphy enables the detection of functional abnormalities, such as increased osteoblastic activity and bone turnover, which frequently precede morphological degeneration.^8^^,^^9^ This capability renders scintigraphy particularly valuable for detecting early or active TMJ-OA. Moreover, the ability of scintigraphy to provide a whole-body overview enables simultaneous assessment of both localized and systemic osteoarthritic involvement, thereby offering a more comprehensive diagnostic perspective in patients with suspected multi-joint disease.

Timely and accurate diagnosis of TMJ-OA is essential for managing symptoms, improving clinical outcomes, and maintaining patients’ quality of life. Radiographic evidence of OA increases with age, with nearly 80% of individuals over 65 showing such signs.^10^ When OA symptoms—such as joint noise, pain, and functional limitations—persist over time, they may contribute to psychological effects, including increased depression and anxiety, as well as social consequences resulting from diminished physical function.^11^ Beyond diagnostic challenges, the underlying pathophysiology of TMJ-OA remains insufficiently understood. It is not yet clear whether the pathological changes are confined to the TMJ and adjacent structures or reflect systemic processes such as chronic inflammation or central nervous system sensitization.^12^ Specifically, it remains uncertain whether TMJ-OA symptoms result solely from localized joint inflammation or are influenced by systemic inflammatory responses and neurophysiological mechanisms. For instance, nearly half of patients with rheumatoid arthritis show signs of TMDs, suggesting a link between localized and systemic joint pathology.^13^ This highlights the need for diagnostic approaches that can account for both local and systemic features of the disease.

Recently, deep learning—particularly convolutional neural networks (CNNs)—has brought major advances to medical image analysis. Unlike conventional machine learning, CNNs can automatically learn relevant features from complex data, enabling strong performance across various imaging modalities.^14^ A recent review reported that artificial intelligence (AI) models perform comparably to human clinicians, with pooled sensitivities of 88% and 80% and specificities of 81% and 79%, respectively.^15^ Among CNN architectures, VGG16 stands out for its balanced design, transfer learning compatibility, and effective feature extraction, making it a popular choice for diagnostic tasks.^16^ Several studies have applied CNNs to TMJ imaging using CBCT or MRI.^17^^,^^18^ However, these modalities are not ideal for identifying early-stage TMJ-OA or monitoring ongoing inflammatory activity. Furthermore, diagnostic interpretations of such images are often subject to inter-observer variability, limiting consistency. In contrast, bone scintigraphy can detect early metabolic changes in the TMJ,^19^ making it a promising modality for early detection. However, the integration of bone scintigraphy into AI-driven diagnostic frameworks for TMJ-OA has not yet been investigated.

**Figure 1:**
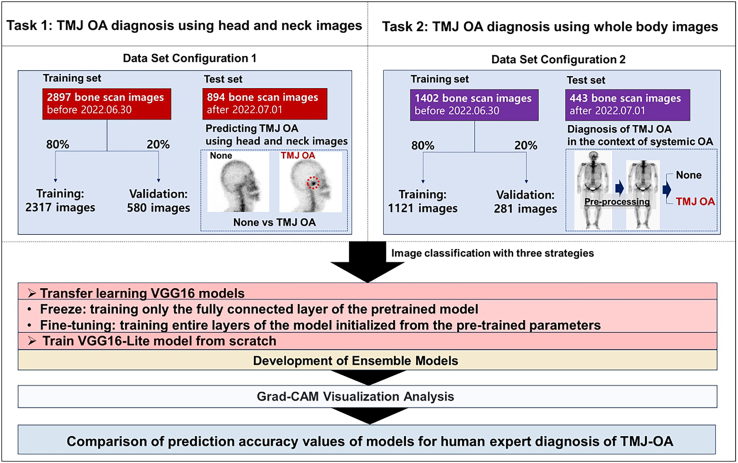
Study protocol Overview of the study protocol, including the dataset and tasks performed for TMJ-OA diagnosis. The dataset consisted of 1,943 TMD patients, with 3,886 TMJs used for analysis.

**Figure 2:**
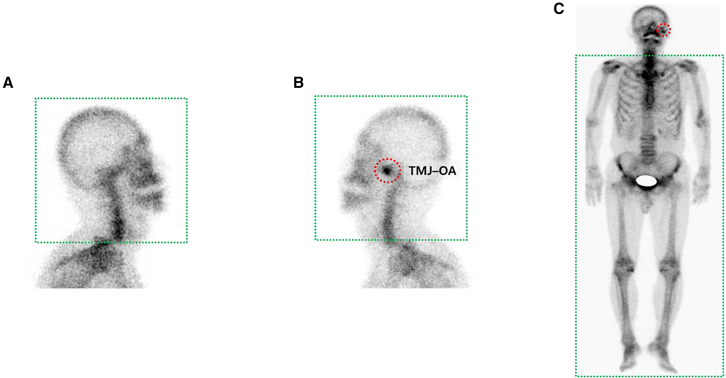
Representative bone scintigraphy images used for TMJ–OA diagnosis and model training (A) Lateral skull view without increased radiotracer uptake in the temporomandibular joint (TMJ) region (non-TMJ-OA). (B) Lateral skull view of the same individual showing increased uptake in the right TMJ (red dotted circle), indicating TMJ osteoarthritis (TMJ-OA). (C) Whole-body anterior bone scan of the same individual, with TMJ-OA site indicated by a red dotted circle. Green dotted boxes represent the regions captured and used as input for deep learning models in task 1 (A and B: head and neck images) and task 2 (C: systemic full-body images).

This study aims to address a critical research gap by developing deep learning models capable of performing automated diagnosis of TMJ-OA using bone scan images (Figure 1). Furthermore, we evaluate whether osteoarthritic changes in other anatomical regions, as visualized through whole-body bone scans, can serve as predictive indicators of TMJ involvement (Figure 2). To this end, we implement three CNN models based on the VGG16 architecture: a freeze model utilizing pretrained weights, a fine-tuned model, and a lightweight VGG16-Lite model trained *de novo*. Ensemble learning strategies are also employed to assess whether combining model outputs can enhance diagnostic robustness and overall performance.

## Results

### Task 1

#### Prediction of TMJ-OA

**Figure 3:**
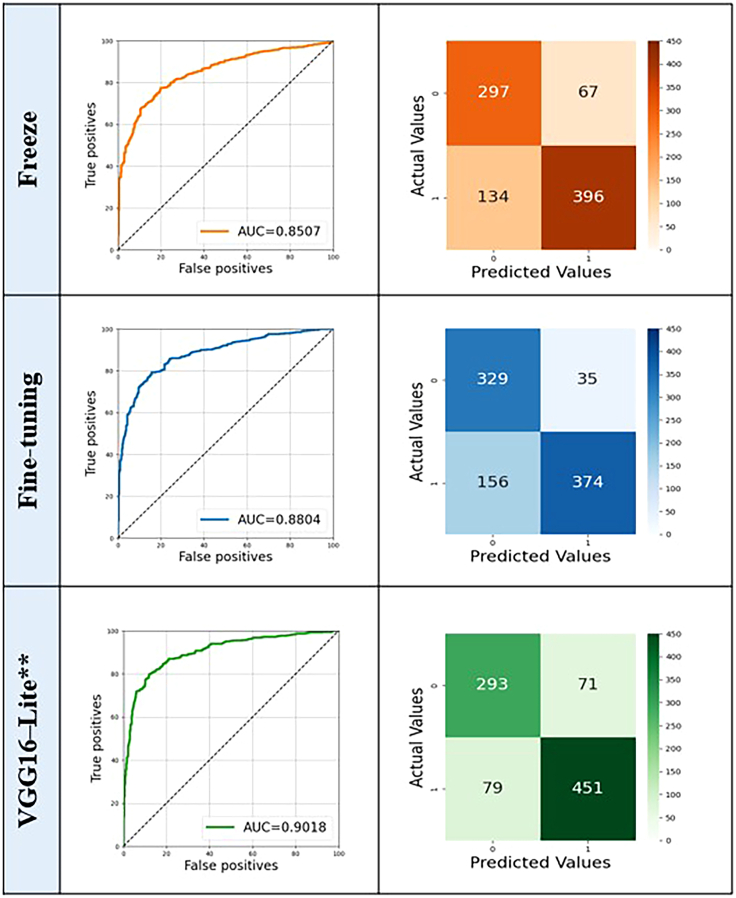
Classification results (ROC curves and confusion matrices) of three CNN models (task 1) Freeze (orange), fine-tuned VGG16 (blue), and VGG16-Lite (green). VGG16-Lite∗∗ shows significantly higher predictive accuracy for TMJ-OA than the other two models (DeLong’s test, two-sided; ∗∗: *p* < 0.01). The area under the receiver operating characteristic curve (AUC) values were 0.9018 (VGG16-Lite), 0.8804 (fine-tuned VGG16), and 0.8507 (Freeze).

In task 1, the VGG16-Lite model demonstrated superior performance (AUC = 0.9018) compared to the fine-tuned (AUC = 0.8804) and freeze (AUC = 0.8507) VGG16 variants (*p* = 0.0043). The optimal operating points for the confusion matrix were determined using Youden’s index, calculated from the validation set. The higher AUC achieved by the VGG16-Lite model suggests that fewer parameters were sufficient for task 1 (Figure 3). Notably, even when more complex models such as VGG19 and EfficientNet were evaluated, they did not yield a significant improvement in AUC compared to VGG16-Lite, supporting the preference for simpler and more efficient models. Nevertheless, all three approaches demonstrated satisfactory performance.

#### Age- and gender-wise TMJ-OA prediction

Because TMJ-OA presentation has been reported to differ by age and gender,^20^^,^^21^ we assessed the model’s suitability across demographic subgroups. The fine-tuned model, which showed higher AUC and prediction accuracy for TMJ-OA than the two transfer-learned VGG models, was selected for comparison with the VGG16-Lite model across different age groups and genders (Table 1). When age was grouped as < 20 years and ≥ 20 years, the VGG16-Lite model achieved significantly higher AUC values than the fine-tuned model in both the < 20 years group (0.8043 vs. 0.8717, *p* = 0.033) and the ≥ 20 years group (0.8708 vs. 0.8901, *p* = 0.032). In a more detailed stratification of the ≥ 20 years group into narrower age bands (20–39, 40–59, and ≥ 60 years), no statistically significant differences in AUC between the two models were observed (*p* > 0.05 for all comparisons).

**Table 1:** Comparison of predictions between the fine-tuned model and VGG16–Lite model for Task 1

Group		Fine-tuning model AUC [95% CI]	VGG16–Lite model AUC [95% CI]	P–value^a^	P–value^b^
Overall	*n* = 894 (100.0%)	0.8804 [0.8579, 0.9019]	0.9018 [0.8811, 0.9212]	0.0043∗∗	–

**Age**					

Under 20	*n* = 147 (16.4%)	0.8043 [0.6837, 0.9045]	0.8717 [0.7576, 0.9636]	0.033∗	0.9949
20–39	*n* = 334 (37.4%)	0.8427 [0.7983, 0.8836]	0.8687 [0.8263, 0.9074]	0.074	
40–59	*n* = 242 (27.1%)	0.8640 [0.8131, 0.9097]	0.8918 [0.8500, 0.9288]	0.125	
Over 60	*n* = 171 (19.1%)	0.8816 [0.8243, 0.9316]	0.8758 [0.8113, 0.9317]	0.799	
Over 20	*n* = 747 (83.6%)	0.8708 [0.8448, 0.8952]	0.8901 [0.8662, 0.9126]	0.032∗	–

**Sex**					

Male	*n* = 248 (27.7%)	0.8932 [0.8502, 0.9313]	0.9077 [0.8671, 0.9435]	0.336	0.9353
Female	*n* = 646 (72.3%)	0.8818 [0.8553, 0.9067]	0.9013 [0.8772, 0.9238]	0.021∗	

Delong’s test was used to compare the two models with the highest and second-highest diagnostic accuracies. Statistical significance was set at a two-tailed *p* value < 0.05. ∗: *p* < 0.05, ∗∗: *p* < 0.01. AUC, area under the curve; CI, Bootstrapped confidence intervals obtained through 100,000 resampling iterations.

a *p* value from comparison between the fine-tuned and VGG16–Lite models.

b *p*-value from comparisons by age group and sex between CNN models yielding the highest diagnostic accuracy. *p* values exceeding 0.05 indicate no significant differences with respect to age group or sex.

To assess potential gender-related effects, we conducted a subgroup analysis comparing AUC values between males and females within each age group. As summarized in Table S1, no statistically significant differences were observed across all age strata (*p* > 0.05 for all), suggesting that the model’s performance is consistent and unbiased across sexes. Given the unequal sample sizes across age groups—particularly the small numbers in adolescent subgroups such as 10–13 years—we employed a non-parametric permutation test to address violations of normality assumptions. Figure S1 displays the bootstrap AUC distributions that support this approach. To further explore age-related heterogeneity in model performance, we conducted pairwise permutation tests across six age subgroups. Although the global test revealed significant overall variation (*p* = 0.0467), none of the pairwise differences remained significant after Holm–Bonferroni correction (Table S2).

By gender, the VGG16-Lite model (AUC = 0.9013, 95% confidence interval (CI): 0.8772–0.9238) demonstrated significantly higher predictive accuracy than the fine-tuned model (AUC = 0.8818, 95% CI: 0.8553–0.9067) in females (*p* = 0.021). Although the absolute AUC values were higher for males than for females, no significant difference was observed between the VGG16-Lite model (AUC = 0.9077, 95% CI: 0.8671–0.9435) and the fine-tuned model (AUC = 0.8932, 95% CI: 0.8502–0.9313; *p* = 0.336).

**Figure 4:**
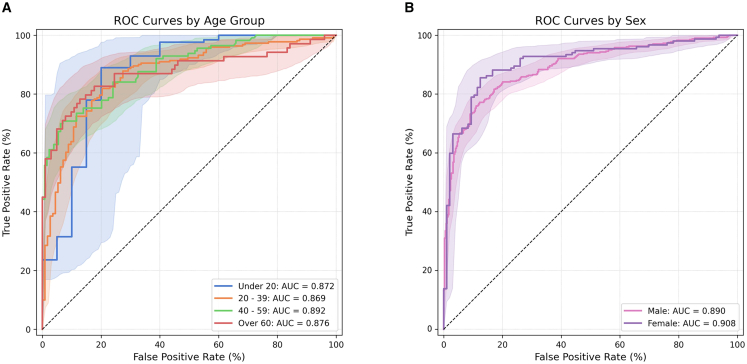
ROC curve comparison of small CNN model according to age and sex groups Receiver operating characteristic (ROC) curve comparison of the VGG16-Lite model across different age and sex groups. (A) Age-wise, the AUC results were as follows: under 20 years, 0.8717; 20–39 years, 0.8687; 40–59 years, 0.8918; and over 60 years, 0.8758. There were no significant differences in AUC values between these age groups (p > 0.05). (B) The VGG16-Lite model achieved the following AUC values: for females, 0.9013 (95% CI: 0.8772–0.9238); for males, 0.9077 (95% CI: 0.8671–0.9435). Statistical significance was assessed using DeLong’s test.-> I only rearranged the sentence order and inserted (A) and (B).

**Figure 5:**
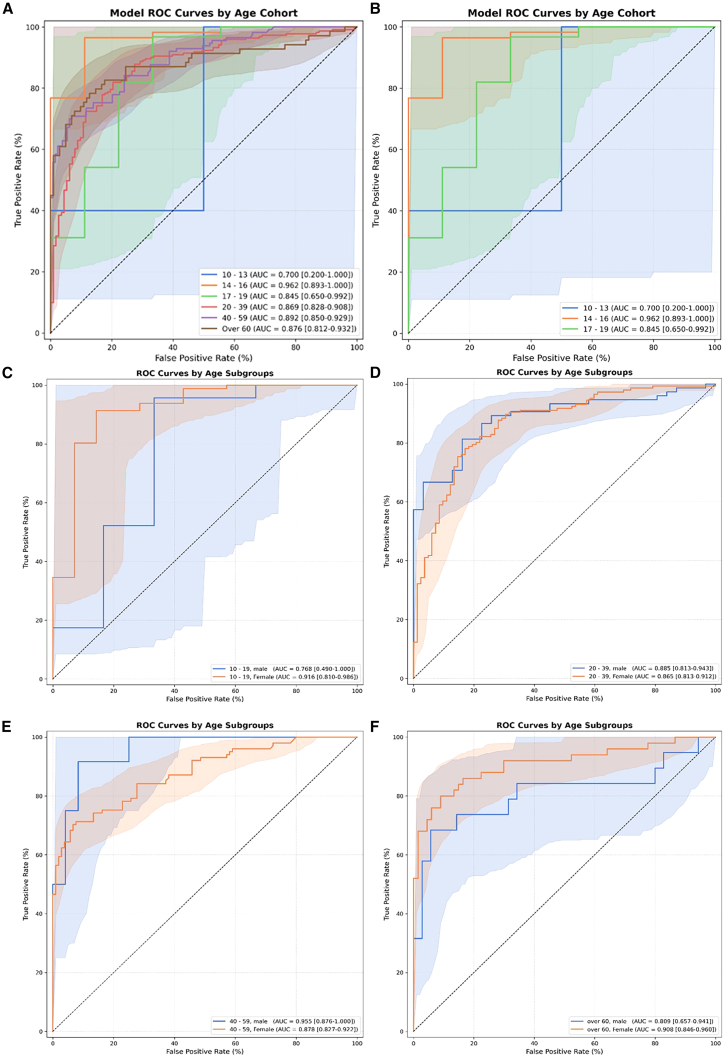
ROC curves of model performance across age and sex subgroups (A) ROC curves by age group: 10–13, 14–16, 17–19, 20–39, 40–59, and over 60 years. No statistically significant differences were observed (permutation test, *p* = 0.0467). (B) ROC curves for adolescent subgroups (10–13, 14–16, 17–19); no significant differences (*p* = 0.0786). (C–F) ROC curves by sex within each age group (10–19, 20–39, 40–59, and over 60 years); no significant differences between males and females (all *p* > 0.1). Statistical significance was assessed using DeLong’s test.

Additionally, in the statistical comparison of AUC values for the VGG16-Lite model across age and gender groups, the *p* values were 0.9949 and 0.9353, respectively, indicating no significant differences in performance by demographic category (Table 1; Figure 4). When evaluating TMJ-OA prediction accuracy by age group, the AUC values were as follows: 40–59 years (0.8918) > over 60 years (0.8758) > under 20 years (0.8717) > 20–39 years (0.8687) (*p* = 0.9949). Although the highest AUC was observed in males, both genders demonstrated outstanding discrimination (AUC >0.9). Overall, task 1 showed no significant variations in diagnostic accuracy by age or gender, and all AUC values fell within the excellent to outstanding range (Figure 5).

#### Grad-CAM visualization

**Figure 6:**
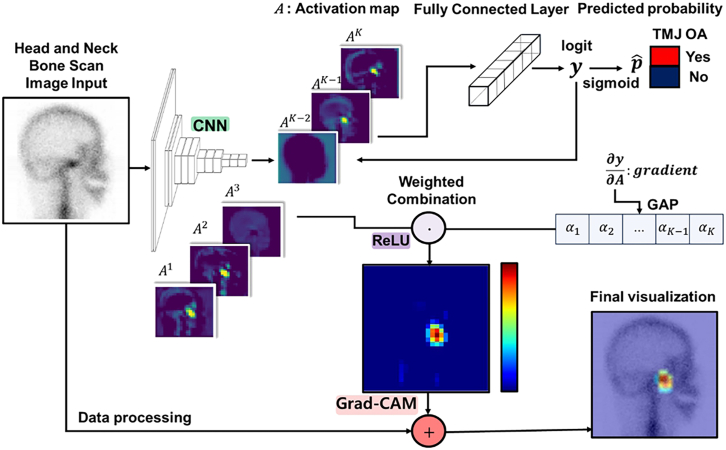
Visualization of TMJ-OA on bone scan images Grad-CAM visualization of the fine-tuned and VGG16-Lite models applied to TMJ-OA bone scan images, showing the region most influential for classification.

Grad-CAM visualization was used to interpret and analyze the learned features of the models (Figure 6). An initial Grad-CAM analysis of feature map activation in the last convolution layer was conducted using true-positive test images, with results from the same images displayed in columns. These images highlight regions of interest targeted by the CNN models. Figure 6 presents a superimposed visualization of three true-positive samples used to compare the results of both models. Each heatmap score was normalized to the range (0, 1) to enhance visibility. While both models identified appropriate regions near the joint, the heatmaps produced by the smaller model highlighted a more localized area.

**Figure 7:**
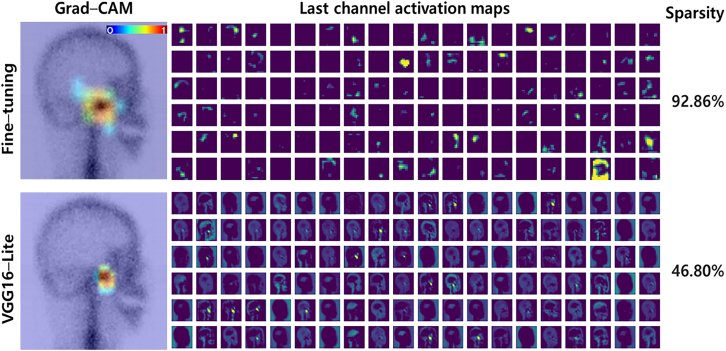
Final activation maps of models using the same image of an OA patient’s side profile Each row shows the results obtained by the two models. Highly sparse activations can be observed in the results obtained by the fine-tuned model.

The activation maps of each model aid in interpreting heatmap differences. In Figure 7, each row displays the results obtained by the fine-tuned and VGG16-Lite models, with 120 activation maps. Sparsity is the percentage of zero values in an activation map. In the Grad-CAM visualizations, the fine-tuned model focused more on joint effusion than on other TMJ structures and showed higher sparsity than the VGG16-Lite model (92.86% vs. 46.80%, *p* < 0.05). Lower sparsity indicates broader neuronal activity, whereas higher sparsity suggests a focus on specific regions with fewer but more relevant activations. This result suggests that the VGG16-Lite model engaged a broader range of TMJ features, in contrast to the localized focus of the fine-tuned model.

### Task 2

#### Prediction with ROC

**Figure 8:**
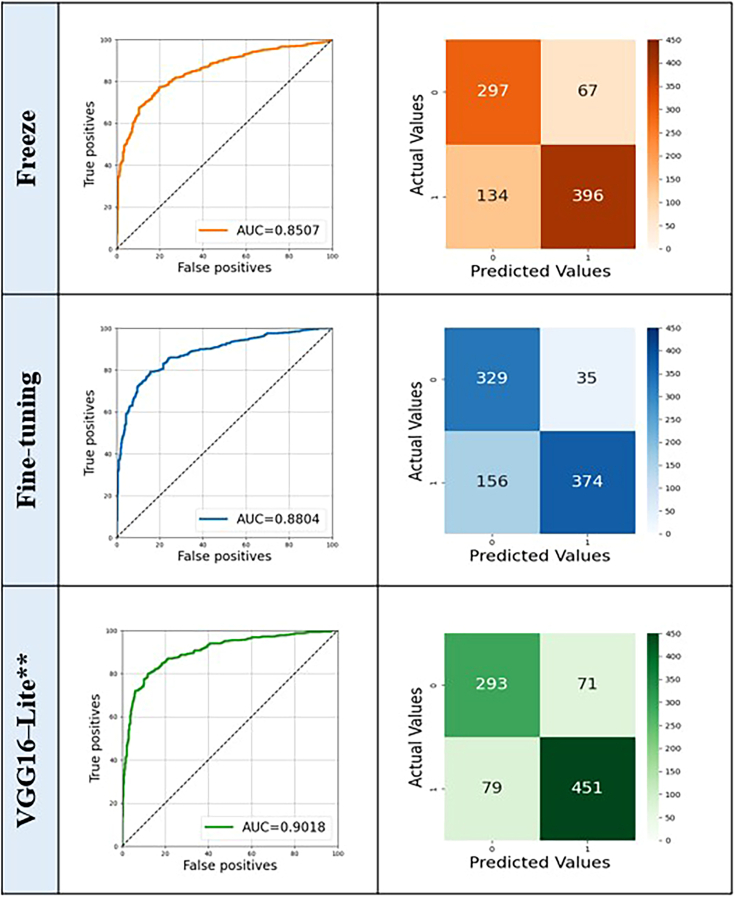
Classification results (ROC curves and confusion matrices) of three CNN models (task 2) ∗ The freeze model had a significantly higher AUC value than the fine-tuned and small CNN models at *p* < 0.05 (DeLong’s test).

Two key findings emerged from task 2, which used whole-body bone scan images excluding the head and neck. First, a reduction in diagnostic accuracy was observed when comparing whole-body images to head and neck images (task 1: VGG-Lite 0.9018 vs. 0.5797; freeze 0.8507 vs. 0.6616; *p* < 0.05). Second, in contrast to task 1, where the VGG16-Lite model outperformed pretrained models, the pretrained models achieved better accuracy than VGG16-Lite in task 2 (*p* = 0.016 and 0.029 for freeze and fine-tuned models, respectively). Specifically, the VGG16-Lite model (AUC = 0.5797) performed worse than the two transfer-learned models, both of which demonstrated acceptable discrimination. No significant difference in AUC was found between the freeze and fine-tuned models (*p* = 0.435). When calculating AUC values for body bone scan images, the freeze model (0.6616) exhibited similar performance to the fine-tuned model (0.6537), with both models significantly outperforming the VGG16-Lite model (*p* = 0.021; Figure 8; Table 2).

**Table 2:** Detailed prediction results of fine-tuned, freeze, and VGG16–Lite models on Task 2

Model	Sensitivity	Specificity	PPV	NPV	Accuracy	AUC [95% CI]	*p* value
Freeze	0.3171	0.8609	0.8667	0.3065	0.4582	0.6616 [0.6037, 0.7181]	0.021^a^
Fine-tuning	0.5549	0.6609	0.8235	0.3423	0.5824	0.6537 [0.5954, 0.7092]	
VGG16–Lite	0.5427	0.5391	0.7706	0.2925	0.5418	0.5797 [0.5197, 0.6371]	

The optimal operating values for accuracy, sensitivity, specificity, positive predictive value (PPV), and negative predictive value (NPV) were determined using Youden’s index as calculated for the overall validation set. Statistical significance was set at *p* < 0.05.

a The freeze model had a significantly higher AUC value than either the fine-tuned or small CNN models at *p* < 0.05 (DeLong’s test).

#### Grad-CAM visualization analysis

**Figure 9:**
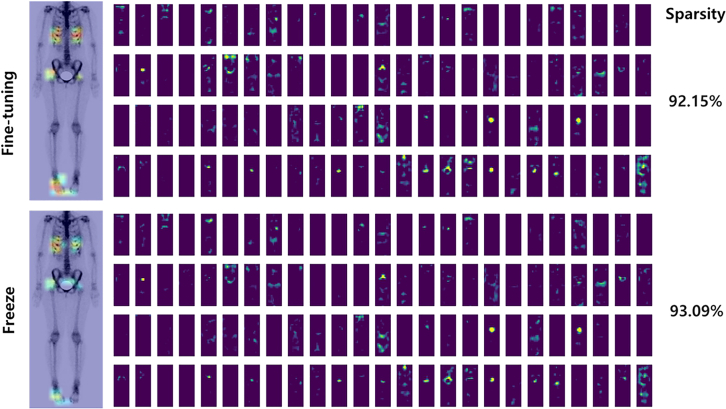
Final activation maps of models using the same body image of a TMJ-OA patient Highly sparse activations are shown for both models.

The final activation maps of the models were examined to elucidate how the fine-tuned model extracted discriminative features of TMJ-OA (Figure 9). Both the VGG16-Lite and fine-tuned VGG16 models demonstrated similar activation patterns and sparsity, suggesting that they learned comparable features. Visual inspection confirmed a high degree of overlap in feature localization, and the correlation coefficient between their prediction outputs was 0.96 (*p* < 0.0001), indicating a strong linear relationship. This similarity supports the rationale for combining these models in an ensemble. TMJ-OA prediction using head and neck bone scans demonstrated outstanding performance, with AUC values exceeding 0.9. Notably, the ensemble of the fine-tuned and VGG16-Lite models achieved an AUC of 0.9012—the highest among all ensemble combinations (Table 3). However, this value was slightly lower than the AUC of the fine-tuned model alone (0.9018), and the difference was not statistically significant (*p* > 0.05). This suggests that ensemble strategies did not substantially enhance diagnostic performance in this context. Importantly, ensembles that included the Freeze model exhibited lower AUCs, likely due to the integration of lower-confidence predictions that diluted the strength of the ensemble. This finding underscores the necessity of judicious model selection in constructing ensemble frameworks. Overall, while ensemble learning may stabilize predictions, its added value depends on the complementarity and individual performance of the component models.

**Table 3:** Ensemble models for Tasks 1 and 2

Task	Ensemble		Sensitivity	Specificity	PPV	NPV	Accuracy	AUC	*p* value
Task 1	Ensemble of two models	Fine-tuning + VGG16–Lite	0.7245	0.9093	0.9209	0.6939	0.7998	0.9012	0.041^a^
		Freeze + VGG16–Lite	0.8358	0.8407	0.8842	0.7786	0.8378	0.8995	
		Fine-tuning + Freeze	0.7302	0.8901	0.9063	0.6938	0.7953	0.8764	
	Ensemble of three models	Fine-tuning + Freeze + VGG16–Lite	0.7472	0.8929	0.9103	0.7081	0.8065	0.8985	
Task 2	Ensemble of two models	Fine-tuning + VGG16–Lite	0.5915	0.6261	0.8186	0.3495	0.6005	0.6563	0.319
		VGG16–Lite	0.2713	0.8870	0.8725	0.2991	0.4312	0.6623	
		Fine-tuning + Freeze	0.3354	0.8261	0.8462	0.3035	0.4628	0.6588	
	Ensemble of three models	Fine-tuning + Freeze + VGG16–Lite	0.6037	0.6174	0.8182	0.3532	0.6072	0.6609	

PPV, positive predictive value; NPV, negative predictive value; AUC, area under the curve; Sensitivity, true positive/true positive + false negative; Specificity, true negative/true negative + false positive; PPV, true positive/true positive + false positive; NPV, true negative/true negative + false negative.

a The fine-tuned + VGG16–Lite ensemble model achieved a significantly higher AUC value than all other ensemble combinations, with statistical significance at *p* < 0.05 (DeLong’s test).

In task 2, where TMJ-OA was predicted using only body bone scan images, the freeze model (AUC = 0.6616) demonstrated similar performance to the fine-tuned model (AUC = 0.6537), and both significantly outperformed the VGG16-Lite model (AUC = 0.5797; *p* = 0.021). No ensemble configuration in task 2 produced a significant improvement over the individual models (*p* = 0.319). The fine-tuned model alone showed the best performance for task 2 (AUC = 0.6537). The AUC values for ensemble models were consistently lower in task 2 than in task 1. Nevertheless, task 2 achieved an acceptable level of diagnostic accuracy, although excluding head and neck data naturally limited the models’ ability to predict TMJ-OA with high precision.

## Discussion

Advancements in AI and deep learning have significantly improved the predictive accuracy, speed, and efficiency of medical diagnostics. Numerous deep-learning models have been developed to assess the risk of systemic OA using baseline X-ray and MRI scans, demonstrating strong diagnostic performance in predicting various OA-related outcomes, including radiographic OA incidence and progression, pain progression, and the need for future treatment or surgery.^22^^,^^23^^,^^24^ Recent AI models utilizing CBCT images for TMJ-OA diagnosis have also shown promising results, with reported accuracies ranging from 86% to 95% when compared with expert radiologist evaluations.^25^^,^^26^ Despite these advances, deep learning research involving bone scans for evaluating TMJ or TMJ-OA remains limited. Conventional morphological imaging techniques—such as X-ray, CT, and MRI—are commonly used for noninvasive bone structure assessment. However, their diagnostic performance for TMJ-OA remains suboptimal, particularly in early-stage detection. According to Smith et al., trained radiologists achieved only 58.7% diagnostic accuracy for knee OA using MRI, whereas deep-learning methods improved this to 65%–72% accuracy.^27^ By analogy, early-stage TMJ-OA may be similarly underdiagnosed or misclassified in clinical settings, underscoring the need for improved diagnostic tools.

While CBCT and MRI provide high-resolution structural information of the TMJ, their diagnostic utility may be limited in early-stage TMJ-OA, where morphological abnormalities are often subtle or absent.^8^^,^^28^ This imaging advantage is particularly relevant in TMJ-OA, where early-stage disease may present without discernible anatomical abnormalities.^29^ In contrast, bone scintigraphy offers complementary insights by capturing early functional changes related to bone metabolism, such as increased osteoblastic activity, through radiotracer uptake.^30^ By enabling the identification of metabolically active lesions before structural changes appear, this functional imaging approach is particularly valuable for the early detection of TMJ-OA, where conventional modalities often fall short. Building upon this strength, the present study explored the application of deep-learning models to bone scan images, aiming to leverage their early-detection potential for the accurate, noninvasive diagnosis of TMJ-OA.

Building upon this strength, the present study focused on the automated prediction of TMJ-OA using bone scan images. The VGG16-Lite model demonstrated excellent predictive performance, achieving an AUC of 90.18%. While ensemble learning is generally used to enhance predictive performance,^31^^,^^32^ our results suggest that its effectiveness depends on model selection. Notably, combining the fine-tuned and VGG16-Lite models yielded a high AUC, whereas inclusion of the underperforming freeze model led to diminished performance. Subgroup analyses indicated that the model maintained excellent to outstanding diagnostic performance across all age and gender groups, suggesting its robustness and broad applicability. Although TMJ-OA is known to present differently across age and gender, our analysis showed no significant performance gap between males and females, and model accuracy was consistent across age groups, indicating generalizability.

Our deep learning model for TMJ-OA demonstrated excellent diagnostic performance overall, with no significant differences observed across most age and sex subgroups. Radiographic studies have reported that the frequency and severity of TMJ-OA lesions tend to rise with age, particularly in the fifth and seventh decades of life.^33^^,^^34^^,^^35^ Older individuals more frequently exhibit bony changes such as condylar flattening and osteophyte formation, even if they report less pain.^36^ These changes are attributed to cumulative mechanical stress, reduced cartilage regenerative capacity, and age-related alterations in joint biology.^37^ Sex-based differences have also been consistently reported, with CBCT-based studies showing that female patients present with a significantly higher prevalence and greater severity of TMJ-OA compared to male patients, potentially due to hormonal and anatomical factors.^38^^,^^39^ Globally, women account for approximately 60% of OA cases, with the sex disparity becoming more pronounced after midlife.^40^ Although sex differences in TMJ-OA have been reported, our analysis suggests sex-independent model performance. Importantly, the model exhibited consistent performance across sexes within each age group, suggesting that it generalizes well without gender-related bias. Future research should pursue the development of personalized diagnostic approaches that incorporate demographic factors to improve the precision and equity of TMJ-OA detection.

This pattern is further supported by the observation that the VGG16-Lite model, which did not rely on pre-trained weights, demonstrated better performance. Smaller or less complex models, such as VGG16-Lite, are generally less prone to overfitting compared to heavier or more complex models, potentially leading to improved performance on test data.^41^ Additionally, the reduction in unnecessary computations increases efficiency, making smaller models advantageous for practical applications.^42^^,^^43^ Although the VGG16-Lite model performed the best for head and neck imaging (task 1), the more complex fine-tuned VGG16 model was more effective for whole-body bone scans (task 2). In more narrowly defined tasks where the TMJ is the focal region, simpler models can avoid overfitting and deliver excellent performance.^44^

However, predicting TMJ-OA from whole-body scans requires the interpretation of non-local features, which may benefit from more complex architectures. While AUC values exceeded 0.9 when using head and neck scans, they dropped to approximately 0.6 for whole-body scans, indicating acceptable but reduced diagnostic accuracy. This pattern reflects that more complex diagnostic tasks involving high-dimensional inputs may require more sophisticated model architectures capable of capturing distributed information effectively.^45^ These results do not necessarily refute a potential association between systemic OA and TMJ-OA. Rather, these findings highlight the limitations of relying solely on systemic OA features as predictors of TMJ-OA. This underscores the importance of exercising caution when generalizing diagnostic predictions across anatomically distinct joint regions. Future studies should further investigate the nature and strength of this relationship through multimodal and longitudinal approaches.

Bone scan imaging is relatively simpler than other imaging modalities. Pathological changes are detected based on the structural arrangement of black-and-white contrasts and the intensity of black regions. Although bone scan images may appear coarse, the diagnostic criteria for OA using these images are well established. In TMJ-OA, a darker region where the temporal bone and mandibular condyle intersect forms a distinct round shape.^46^ Given the lower complexity of bone scan images, they lend themselves well to lightweight AI architectures capable of maintaining high diagnostic accuracy.

In our visualizations, higher sparsity—defined as a state in which fewer neurons are activated and more are suppressed—did not correlate with improved diagnostic accuracy. This finding contrasts with prior MRI-based studies, in which models exhibiting high sparsity demonstrated superior performance in detecting TMJ abnormalities such as anterior disc displacement and effusion.^32^^,^^47^ The discrepancy may stem from fundamental differences between the imaging modalities. MRI provides high-resolution structural detail, making it well suited to sparsity-driven attention mechanisms that emphasize focal pathology.^48^ In contrast, bone scans offer lower spatial resolution but capture functional information over broader anatomical regions.^9^^,^^49^ In this context, diagnostic performance may depend less on overall sparsity and more on a model’s ability to focus on regions that correspond to disease-relevant pathological changes. Interestingly, in the case of whole-body bone scans—where multiple focal sites of uptake are often present—models with very high sparsity levels (>90%) appeared to perform well, potentially due to their capacity to selectively isolate metabolically active regions.^50^

Despite their theoretical advantages, ensemble models do not always lead to improved performance over individual models. Contrary to expectations, combining two or three deep-learning models did not yield a higher AUC for TMJ-OA prediction in our study. For example, Pi et al. reported that ensemble networks used to diagnose knee OA from MRI often misclassified early-stage pathology as either normal or advanced.^51^ The primary motivation for ensembling different models in AI algorithm development is to improve accuracy by combining mutually complementary models, with each model excelling in areas where others may not.^52^ Nevertheless, ensemble strategies do not always guarantee improved outcomes. When constituent models share similar architectural structures or extract overlapping features, the ensemble may lack sufficient diversity and, in some cases, introduce noise rather than enhance predictive performance.^53^ This outcome may be attributed to the architectural similarity and feature redundancy among the component models, highlighting the importance of model diversity in ensemble strategies. Our results revealed that when diagnosing TMJ-OA from head and neck images, all individual models heavily focused on the TMJ, demonstrating reliance on similar feature sets. Moreover, because we did not include models with fundamentally distinct learning strategies or architectures—such as decision trees, transformers, or attention-based networks—the ensemble lacked the necessary heterogeneity to provide a broader and more nuanced interpretation of the data.^54^ Future research should focus on building ensemble frameworks that integrate models based on heterogeneous architectures or multiple imaging modalities, along with relevant clinical variables.

To conclude, this study demonstrated the feasibility of using deep learning models, particularly the VGG16-Lite architecture, for the automatic diagnosis of TMJ-OA based on bone scan images. The model achieved high diagnostic accuracy with minimal computational cost, highlighting its clinical utility in resource-limited settings. TMJ-focused imaging outperformed whole-body scans, emphasizing the importance of targeted approaches. Future studies should validate these findings across diverse populations and explore multimodal and personalized diagnostic frameworks.

### Limitations of the study

This study faced several limitations. The models were developed using data from a single institution, potentially increasing the risk of overfitting. Future multicenter studies are needed to validate these findings. TMDs have multifactorial etiologies and show considerable variation in etiology, pathophysiology, and clinical characteristics by age, gender, and life stage.^55^^,^^56^ The incidence of systemic OA increases notably after the age of 55, and its clinical presentation differs between males and females.^57^ However, few studies have addressed TMJ-OA diagnosis in relation to age and gender, suggesting a need for datasets with more balanced demographic representation. Our decision to analyze model performance by age and gender adds valuable insight. To better understand the causal relationship and mutual influence between systemic OA and TMJ-OA, future research should incorporate carefully designed experiments and diverse datasets. Additionally, although this study relied exclusively on bone scan images, incorporating CBCT or CT scans in future work could enhance diagnostic robustness and provide further validation of the model’s effectiveness.

## Resource availability

### Lead contact

Further information and requests for resources and materials should be directed to and will be fulfilled by the lead contact, Prof. Yeon-Hee Lee (omod0209@gmail.com).

### Materials availability

This study did not generate new unique reagents or materials.

### Data and code availability

#### Data

The full dataset of bone scan images associated with this publication is available at a publicly accessible repository (https://github.com/aspro509/Bonescan).

#### Code

All associated code needed to reproduce the results in this publication has been deposited in a publicly accessible repository (https://github.com/aspro509/Bonescan).

#### Additional information

Any additional information required to reanalyze the data reported in this article is available from the lead contact upon request.

## Acknowledgments

The authors extend special thanks to Sung–Woo Lee of the Department of Oral Medicine and Oral Diagnosis at 10.13039/501100002551Seoul National University. This work was supported by the 10.13039/501100003725National Research Foundation of Korea (10.13039/100028114NRF) grant funded by the Korean Government (MSIT) (no. RS-2024-00421203), the Institute of Information & Communications Technology Planning & Evaluation (IITP) grant funded by the MSIT (IITP-2021-0-02068, RS-2020-II201373, RS-2023-00220628), and a research grant from 10.13039/501100002597Kyung Hee University in 2025 (KHU-20251299).

## Author contributions

Y.-H.L., H.-S.K., and A.C. wrote the manuscript. Y.-H.L., Q.-S.A., S.C., and I.K.H. contributed to the data acquisition. Y.-H.L., H.-S.K., and S.J. contributed to the data analysis. Y.-H.L., H.-S.K., and S.J. contributed to the data interpretation. Y.-H.L., Y.-K.N., G.F.P.S., and A.C. provided the expertise. Y.-H.L. and H.-S.K. prepared to the figures. Y.-H.L., I.K.H., Y.-K.N., and A.C. provided their expertise and contributed to the revisions. All the authors have read and agreed to the published version of this manuscript.

## Declaration of interests

The authors declare that they have no competing interests.

## STAR★Methods

### Key resources table

**Table:** 

REAGENT or RESOURCE	SOURCE	IDENTIFIER
**Deposited data**		

Human bone scintigraphy scans (TMJ and whole-body)	Kyung Hee University Dental Hospital	https://github.com/aspro509/Bonescan
TMJ bone scintigraphy dataset (patient-level data)	This paper	https://github.com/aspro509/Bonescan

**Software and algorithms**		

Python v3.10.12	Python Software Foundation	https://www.python.org
PyTorch (deep learning framework)	Meta AI	https://pytorch.org
Optuna (hyperparameter optimization)	Preferred Networks	https://optuna.org
IBM SPSS Statistics v26	IBM Corp.	https://www.ibm.com/spss
R software v4.0.2	R Foundation	https://cran.r-project.org
Custom analysis code (this paper)	–	https://github.com/aspro509/Bonescan

**Other**		

Siemens ECAM gamma camera	Siemens Healthineers	Model: ECAM
Siemens Symbia dual-head gamma camera	Siemens Healthineers	Model: Symbia

### Experimental model and study participant details

The study protocol was approved by the Institutional Review Board of Kyung Hee University Dental Hospital in Seoul, South Korea (KHD IRB, IRB No.: KH–DT23–057–001), in accordance with the principles of the Declaration of Helsinki. Informed consent was obtained from all participants.

A total of 1,943 TMD patients (555 male and 1,388 female, representing 3,886 TMJs), aged 10–92 years (mean age: 40.32 ± 19.35 years), who were clinically diagnosed with TMD and visited Kyung Hee University Dental Hospital between January 1, 2019, and May 31, 2023, were included in this study. All participants underwent comprehensive bone scintigraphy. Of the 3,886 TMJs, 2,148 (55.28%) were diagnosed with TMJ–OA, whereas 1,738 (44.72%) were classified as non–TMJ–OA, including cases with OA in other body regions. All participants were of a single ethnicity, Asian (Korean).

This study consisted of two tasks. Task 1 involved diagnosing TMJ–OA from head and neck bone scan images, and Task 2 focused on predicting TMJ–OA using whole-body bone scan images. For Task 1, the entire dataset (3,886 TMJs from 1,943 patients) was divided into training and testing sets using an 80:20 ratio based on the patients’ visit dates to the hospital. Specifically, 2,897 head and neck bone scan images from 1,449 patients who visited the hospital between May 2019 and June 2022 comprised the training set, and 894 images from 447 patients who visited between July 2022 and May 2023 comprised the testing set. The training set was further split into an 80:20 ratio, with 80% used to train the AI models and 20% reserved for validation. For Task 2, images from 923 of the 1,943 TMD patients were used to build the model. Among the patients included in Task 1, 1,020 who had head and neck bone scan images but did not undergo whole-body bone scans were excluded from Task 2. These images were also divided into training and testing sets using the previously described ratio (Figure 1).

### Method details

#### Diagnostic criteria for TMJ–OA

Notably, the diagnosis of TMJ–OA requires both clinical examination and imaging evaluation of the joint.^58^ In this study, TMJ–OA was diagnosed using a two-step approach that combined radiologic imaging and clinical evaluation. First, qualitative and quantitative assessments of bone scintigraphy were independently conducted by two board-certified nuclear medicine specialists (IKH and SC; Kappa = 0.82–0.87), in accordance with the American College of Rheumatology criteria for osteoarthritis diagnosis.^8^^,^^59^ Increased uptake in the TMJ region on bone scans was interpreted as reflecting active bone remodeling or inflammation associated with osteoarthritic changes. In the qualitative analysis, the simple uptake level of 99mTc–hydroxymethylene diphosphonate (99mTc–HDP) by each TMJ was visually assessed relative to adjacent structures, including the ipsilateral and contralateral parietal bones and contralateral TMJ. Uptake was considered positive for TMJ–OA when the TMJ signal was higher than that of the surrounding bone or contralateral TMJ. If no increased uptake was observed bilaterally, the scan was labeled as negative. For quantitative analysis, regions of interest (13 × 13 pixels) were selected on both the TMJ and parietal bone areas to calculate uptake ratios, thereby supporting the visual interpretations. Any disagreements between the two radiologists were resolved through discussion until consensus was reached. Subsequently, two experienced TMD specialists (YHL and QSA) determined the final diagnostic label for each case based on the Diagnostic Criteria for Temporomandibular Disorders (DC/TMD) Axis I.^60^ According to these criteria, a diagnosis of TMJ–OA was considered when clinical signs such as joint sounds—particularly fine or coarse crepitus—were present during mandibular movement, as confirmed by palpation or auscultation.^60^ Additionally, patients typically reported TMJ-related pain, either spontaneously or during functional jaw activities. These clinical findings were supported by radiographic evidence of degenerative changes in the TMJ, including increased radiotracer uptake on bone scintigraphy. The TMD specialists conducted a comprehensive clinical evaluation, incorporating patient history, physical examination, clinical reports, panoramic radiographs, and the aforementioned bone scan results. The inter-rater agreement between the two specialists was high (Kappa = 0.84–0.88), and any diagnostic discrepancies were resolved through discussion. This combined diagnostic workflow ensured that both symptom-based and imaging-based criteria were satisfied, thereby enhancing the validity and reproducibility of the TMJ–OA dataset.

Patients were excluded if they had a history of serious injuries (e.g., unstable multiple traumas to the orofacial region), maxillary or mandibular fractures, pregnancy, psychological conditions, psychiatric or neurological disorders, active bone lesions from causes unrelated to systemic OA, or if TMJ–OA could not be clearly confirmed or ruled out based on bone scan images.^32^

#### Bone scan image acquisition

Bone scintigraphy was performed on 3,886 TMJs from 1,943 symptomatic TMD patients. ^99m^Tc–hydroxymethylene diphosphonate was administered intravenously at a dose of 740 MBq × body weight/70 kg.^8^ Images were acquired 3 h post-injection using dual-head gamma cameras (ECAM and Symbia; Siemens, Erlangen, Germany) equipped with low-energy high-resolution collimators. Whole-body anterior and posterior images were captured using a 256 × 1,024 matrix. Bilateral TMJ images were acquired using a 256 × 256 matrix, with a count density of 200,000 per image.

#### Preprocessing of bone scan images

This study involved two distinct predictive tasks: Task 1—detecting TMJ–OA using head and neck bone scan images; and Task 2—predicting TMJ–OA based on osteoarthritic changes observed in whole-body bone scan images excluding the TMJ region. To enable accurate model training, all bone scan images underwent a standardized preprocessing pipeline. After acquiring the bone scan images for Task 1, noise reduction was performed using a Gaussian filter, and the region of interest surrounding the TMJ was segmented from the original images. All images were resized and optimized to 224 × 224 pixels and converted into three-channel images, where each pixel included intensity values for the red, green, and blue channels. This formatting was necessary to meet the input requirements of a pretrained VGG model, with final input dimensions of 224 × 224 × 3. Several data augmentation techniques were applied to enhance dataset diversity and model robustness. These included random brightness adjustments to simulate varying lighting conditions, random rotations at 10° intervals, spatial translations, and affine transformations to modify the geometric structure of the images. These augmentations improved model generalization by introducing a wider range of image variations (Figures 2A and 2B).

For Task 2, unnecessary regions in the whole-body bone scan images, including empty spaces and areas above the shoulders, were removed. Subsequently, to investigate whether OA in other body joints could be used as a predictor of TMJ–OA, the TMJs in the head and neck regions were excluded during preprocessing (Figure 2C). The images were then resized and optimized to 608 × 224 pixels and converted into a three-channel format, resulting in input dimensions of 608 × 224×3. Bone scan images were labeled as positive if the patient had TMJ-OA on either side and negative if the patient had no TMJ–OA. The same techniques used in Task 1 were employed to enhance dataset diversity and perform data augmentation.

#### Training schemes and interpretation of TMJ–OA

The AI models in this study were implemented using PyTorch and trained on a single A100 graphical processing unit. All models used an effective batch size of 64, adjusted for gradient accumulation. Hyperparameters (e.g., learning rate, loss function, and hidden layer size) were optimized using Optuna,^61^ with 100 optimization trials conducted for each model. After optimization, each model was trained for 100 epochs to produce final versions for evaluation.

The VGG16 model was chosen for its clear architecture, high area under the curve (AUC) scores, and suitability for analyzing learned features and activation maps. For Task 1, three variants of the VGG16 model were used: a freeze model, a fine-tuned model, and a VGG16–Lite model trained from scratch. The freeze model used a pretrained VGG16 with all layers frozen except for the fully connected layers, preventing weight updates in most of the network. In the fine-tuned model, several of the upper convolutional layers were reactivated and trained at a reduced learning rate. Both models follow the standard VGG16 architecture, which includes 13 convolutional layers, five max-pooling layers, and three fully connected layers. Although the freeze model retained most of the pretrained weights, the fine-tuned model selectively updated the higher convolutional layers.

The VGG16–Lite model, by contrast, was designed for greater computational efficiency. It consists of only six convolutional layers and 288,929 parameters, compared to the 13 layers and 17.92 million parameters in the standard VGG16, enabling faster inference. The VGG16–Lite model was trained from scratch to evaluate whether a simplified architecture could still accurately predict TMJ–OA. It includes convolutional layers with rectified linear unit activation, max pooling, batch normalization, and a fully connected layer. The learning rates for the freeze, fine-tuned, and VGG16–Lite models were set to 1e^−3^, 1e^−6^, and 1e^−3^, respectively, using the Adam optimizer and early stopping based on validation AUC values, in accordance with prior studies.^47^^,^^62^

For Task 2, the same three approaches (i.e., freeze, fine-tuned, and VGG16–Lite) were used to predict TMJ–OA from whole-body bone scan images. The respective learning rates were 5e^−4^, 1e^−5^, and 1e^−3^, with the Adam optimizer and early stopping based on the validation AUC. Additionally, ensemble methods were evaluated by combining predictions from pairs or all three models to determine whether such combinations improved diagnostic accuracy. The AUC values from these ensemble models were recorded to assess their overall performance.

#### Grad–CAM visualization

Gradient-weighted class activation mapping (Grad-CAM) was used to interpret and analyze the learned features of the fine-tuned and VGG16–Lite models by comparing identical images that both models accurately predicted as positive. A Grad–CAM analysis of the feature map activation of the last convolution layer was performed using the test images (A^k^: *k*th activation map). The Grad–CAM heatmap *L*^*c*^ was acquired as follows:

$$L_{Grad-CAM}^{c}=ReLU\left(\sum_{k}\alpha_{k}^{c}A^{k}\right),\alpha_{k}^{c}=\frac{1}{Z}\sum_{i}\sum_{j}\;\frac{\partial y^{c}}{\partial A_{ij}^{k}}$$

After combining the heatmap with the input image, the results highlighted the most critical regions of the bone scan image for prediction (Figure 3). Grad–CAM illustrates the key area of the input image when it is time to predict a label by calculating the importance weight $\alpha_{k}^{c}$, represented by the average value $\frac{\partial y^{c}}{\partial A^{k}}$ where *y*^*c*^ is the logit of class c and *A*^*k*^is the *k*th activation map. Applying a heatmap *L*^*c*^ to the input image illustrates the key area for prediction, as the heatmap visualizes the pixels that most significantly affect *y*^*c*^. We present the Grad-CAM images for visualization.

#### Ensemble model

Several ensemble configurations were used to evaluate potential improvements in predictive performance over that of single AI models. Combinations of the freeze, fine-tuned, and VGG16–Lite models were trained using bone scan images, and the pairs of predicted values were averaged. The following probability equations were used for the ensemble models:

$$\dot{\tilde{P}}=\frac{1}{2}\left(P_{1}+P_{2}\right)\;or\;\frac{1}{3}\;\left(P_{1}+P_{2}+P_{3}\right)$$

where *P1, P2,* and *P3* were predicted from the two (freeze + fine-tuned, freeze + VGG16–Lite, or fine-tuned + VGG16–Lite) or three (freeze + fine-tuned + VGG16–Lite) AI models, respectively. Each model was trained on the augmented datasets of bone scan images. When all three models were combined in an ensemble, the probability value was obtained using the following equation:

$$\hat{p}=\frac{1}{Z}\sum_{i}{\hat{p}}_{i}$$

#### Code availability

The code for the AI algorithm developed in this study for diagnosing TMJ-OA in patients with TMD based on bone scan images is available on GitHub at https://github.com/aspro509/Bonescan.

### Quantification and statistical analysis

Descriptive statistics are reported as means ± standard deviations or as frequencies with percentages. To analyze the distribution of categorical data, we used χ^2^ tests for equality of proportions, Fisher’s exact tests, and Bonferroni tests. All statistical analyses were conducted using IBM SPSS Statistics for Windows (version 26.0; IBM Corp., Armonk, NY, USA), R Version 4.0.2 (R Foundation for Statistical Computing, Vienna, Austria), and Python Version 3.10.12 (Python Software Foundation, DE, USA). ROC curves were plotted, and the area under the curve (AUC) was calculated for each model. AUC = 0.5 indicates no discrimination, 0.6 ≥ AUC >0.5 indicates poor discrimination, 0.7 ≥ AUC >0.6 indicates acceptable discrimination, 0.8 ≥ AUC >0.7 indicates excellent discrimination, and AUC >0.9 indicates outstanding discrimination.^63^ DeLong’s test was used to compare the AUC values for each pair of AI models. Analysis of variance and post hoc tests were conducted to compare the mean AUC values of the three prediction models. McNemar’s test was used to compare the prediction accuracy of the CNN models against that of human experts. Statistical significance was defined as a two-tailed *p*-value <0.05.

### Additional resources

Additional methodological guidance and reproducibility resources are available from the corresponding author upon reasonable request.

## References

[1] Kalladka M., Quek S., Heir G., Eliav E., Mupparapu M., Viswanath A. (2014). Temporomandibular joint osteoarthritis: diagnosis and long-term conservative management: a topic review. J. Indian Prosthodont. Soc..

[2] Lee Y.H., Park H.K., Auh Q.S., Nah H., Lee J.S., Moon H.J., Heo D.N., Kim I.S., Kwon I.K. (2020). Emerging Potential of Exosomes in Regenerative Medicine for Temporomandibular Joint Osteoarthritis. Int. J. Mol. Sci..

[3] Song H.J., Choi H.M., Shin B.M., Kim Y.J., Park M.S., Kim C. (2024). Age-stratified analysis of temporomandibular joint osteoarthritis using cone-beam computed tomography. Imaging Sci. Dent..

[4] Wang X.D., Zhang J.N., Gan Y.H., Zhou Y.H. (2015). Current understanding of pathogenesis and treatment of TMJ osteoarthritis. J. Dent. Res..

[5] MacDonald D., Telyakova V. (2024). An Overview of Cone-Beam Computed Tomography and Dental Panoramic Radiography in Dentistry in the Community. Tomography.

[6] Lee Y.H., Lee K.M., Auh Q.S., Hong J.P. (2017). Magnetic Resonance Imaging-Based Prediction of the Relationship between Whiplash Injury and Temporomandibular Disorders. Front. Neurol..

[7] Bag A.K., Gaddikeri S., Singhal A., Hardin S., Tran B.D., Medina J.A., Curé J.K. (2014). Imaging of the temporomandibular joint: An update. World J. Radiol..

[8] Lee Y.H., Hong I.K., Chun Y.H. (2019). Prediction of painful temporomandibular joint osteoarthritis in juvenile patients using bone scintigraphy. Clin. Exp. Dent. Res..

[9] Epstein J.B., Rea A., Chahal O. (2002). The use of bone scintigraphy in temporomandibular joint disorders. Oral Dis..

[10] Katz J.N., Arant K.R., Loeser R.F. (2021). Diagnosis and Treatment of Hip and Knee Osteoarthritis: A Review. JAMA.

[11] Lee Y., Lee S.H., Lim S.M., Baek S.H., Ha I.H. (2020). Mental health and quality of life of patients with osteoarthritis pain: The sixth Korea National Health and Nutrition Examination Survey (2013-2015). PLoS One.

[12] Li B., Guan G., Mei L., Jiao K., Li H. (2021). Pathological mechanism of chondrocytes and the surrounding environment during osteoarthritis of temporomandibular joint. J. Cell Mol. Med..

[13] Mustafa M.A., Al-Attas B.A., Badr F.F., Jadu F.M., Wali S.O., Bawazir Y.M. (2022). Prevalence and Severity of Temporomandibular Disorders in Rheumatoid Arthritis Patients. Cureus.

[14] Sarker I.H. (2021). Deep Learning: A Comprehensive Overview on Techniques, Taxonomy, Applications and Research Directions. SN Comput. Sci..

[15] Mohammadi S., Salehi M.A., Jahanshahi A., Shahrabi Farahani M., Zakavi S.S., Behrouzieh S., Gouravani M., Guermazi A. (2024). Artificial intelligence in osteoarthritis detection: A systematic review and meta-analysis. Osteoarthritis Cartilage.

[16] Yadav S.S., Jadhav S.M. (2019). Deep convolutional neural network based medical image classification for disease diagnosis. J. Big Data.

[17] Lim N.H., Wen C., Vincent T.L. (2020). Molecular and structural imaging in surgically induced murine osteoarthritis. Osteoarthr. Cartil..

[18] Fan X., Sun A.R., Young R.S.E., Afara I.O., Hamilton B.R., Ong L.J.Y., Crawford R., Prasadam I. (2024). Spatial analysis of the osteoarthritis microenvironment: techniques, insights, and applications. Bone Res..

[19] Van den Wyngaert T., Strobel K., Kampen W.U., Kuwert T., van der Bruggen W., Mohan H.K., Gnanasegaran G., Delgado-Bolton R., Weber W.A., Beheshti M. (2016). The EANM practice guidelines for bone scintigraphy. Eur. J. Nucl. Med. Mol. Imaging.

[20] Yadav S., Yang Y., Dutra E.H., Robinson J.L., Wadhwa S. (2018). Temporomandibular Joint Disorders in Older Adults. J. Am. Geriatr. Soc..

[21] Bagis B., Ayaz E.A., Turgut S., Durkan R., Özcan M. (2012). Gender difference in prevalence of signs and symptoms of temporomandibular joint disorders: a retrospective study on 243 consecutive patients. Int. J. Med. Sci..

[22] Kijowski R., Fritz J., Deniz C.M. (2023). Deep learning applications in osteoarthritis imaging. Skelet. Radiol..

[23] Eckstein F., Chaudhari A.S., Fuerst D., Gaisberger M., Kemnitz J., Baumgartner C.F., Konukoglu E., Hunter D.J., Wirth W. (2022). Detection of Differences in Longitudinal Cartilage Thickness Loss Using a Deep-Learning Automated Segmentation Algorithm: Data From the Foundation for the National Institutes of Health Biomarkers Study of the Osteoarthritis Initiative. Arthritis Care Res..

[24] Ahmed R., Imran A.S. (2024). Knee Osteoarthritis Analysis Using Deep Learning and XAI on X-Rays. IEEE Access.

[25] Ou J., Zhang J., Alswadeh M., Zhu Z., Tang J., Sang H., Lu K. (2025). Advancing osteoarthritis research: the role of AI in clinical, imaging and omics fields. Bone Res..

[26] Lee K.S., Kwak H.J., Oh J.M., Jha N., Kim Y.J., Kim W., Baik U.B., Ryu J.J. (2020). Automated Detection of TMJ Osteoarthritis Based on Artificial Intelligence. J. Dent. Res..

[27] Schiratti J.B., Dubois R., Herent P., Cahané D., Dachary J., Clozel T., Wainrib G., Keime-Guibert F., Lalande A., Pueyo M. (2021). A deep learning method for predicting knee osteoarthritis radiographic progression from MRI. Arthritis Res. Ther..

[28] Samuel A.M., Jain H. (2012). Scintigraphic changes of osteoarthritis: An analysis of findings during routine bone scans to evaluate the incidence in an Indian population. Indian J. Nucl. Med..

[29] Ko Y.Y., Yang W.F., Leung Y.Y. (2024). The Role of Cone Beam Computed Tomography (CBCT) in the Diagnosis and Clinical Management of Medication-Related Osteonecrosis of the Jaw (MRONJ). Diagnostics.

[30] Ulmert D., Solnes L., Thorek D.L. (2015). Contemporary approaches for imaging skeletal metastasis. Bone Res..

[31] Naderalvojoud B., Hernandez-Boussard T. (2023). Improving machine learning with ensemble learning on observational healthcare data. AMIA Annu. Symp. Proc..

[32] Lee Y.H., Won J.H., Kim S., Auh Q.S., Noh Y.K. (2022). Advantages of deep learning with convolutional neural network in detecting disc displacement of the temporomandibular joint in magnetic resonance imaging. Sci. Rep..

[33] Steinmetz J.D., Culbreth G.T., Haile L.M., Rafferty Q., Lo J., Fukutaki K.G., Cruz J.A., Smith A.E., Vollset S.E., Brooks P.M. (2023). Global, regional, and national burden of osteoarthritis, 1990–2020 and projections to 2050: a systematic analysis for the Global Burden of Disease Study 2021. Lancet Rheumatol..

[34] Shane Anderson A., Loeser R.F. (2010). Why is osteoarthritis an age-related disease?. Best Pract. Res. Clin. Rheumatol..

[35] Alzahrani A., Yadav S., Gandhi V., Lurie A.G., Tadinada A. (2020). Incidental findings of temporomandibular joint osteoarthritis and its variability based on age and sex. Imaging Sci. Dent..

[36] Ottersen M.K., Abrahamsson A.K., Larheim T.A., Arvidsson L.Z. (2019). CBCT characteristics and interpretation challenges of temporomandibular joint osteoarthritis in a hand osteoarthritis cohort. Dentomaxillofac. Radiol..

[37] Buckwalter J.A., Anderson D.D., Brown T.D., Tochigi Y., Martin J.A. (2013). The Roles of Mechanical Stresses in the Pathogenesis of Osteoarthritis: Implications for Treatment of Joint Injuries. Cartilage.

[38] Larheim T.A., Abrahamsson A.K., Kristensen M., Arvidsson L.Z. (2015). Temporomandibular joint diagnostics using CBCT. Dentomaxillofac. Radiol..

[39] Almpani K., Tran H., Ferri A., Hung M. (2023). Assessment of condylar anatomy and degenerative changes in temporomandibular joint disorders – A scoping review. J. Oral Biol. Craniofac. Res..

[40] Segal N.A., Nilges J.M., Oo W.M. (2024). Sex differences in osteoarthritis prevalence, pain perception, physical function and therapeutics. Osteoarthr. Cartil..

[41] Gygi J.P., Kleinstein S.H., Guan L. (2023). Predictive overfitting in immunological applications: Pitfalls and solutions. Hum. Vaccin. Immunother..

[42] Ali S., Abuhmed T., El-Sappagh S., Muhammad K., Alonso-Moral J.M., Confalonieri R., Guidotti R., Del Ser J., Díaz-Rodríguez N., Herrera F. (2023). Explainable Artificial Intelligence (XAI): What we know and what is left to attain Trustworthy Artificial Intelligence. Inf. Fusion.

[43] Tan M., Le Q., Kamalika C., Ruslan S. (2019). Proceedings of the 36th International Conference on Machine Learning.

[44] Xu C., Coen-Pirani P., Jiang X. (2023). Empirical Study of Overfitting in Deep Learning for Predicting Breast Cancer Metastasis. Cancers (Basel).

[45] Gosiewska A., Kozak A., Biecek P. (2021). Simpler is better: Lifting interpretability-performance trade-off via automated feature engineering. Decis. Support Syst..

[46] Kim J.H., Kim Y.K., Kim S.G., Yun P.Y., Kim J.D., Min J.H. (2012). Effectiveness of bone scans in the diagnosis of osteoarthritis of the temporomandibular joint. Dentomaxillofac. Radiol..

[47] Lee Y.-H., Jeon S., Won J.-H., Auh Q.S., Noh Y.-K. (2024). Automatic detection and visualization of temporomandibular joint effusion with deep neural network. Sci. Rep..

[48] Kumar R., Pallagatti S., Sheikh S., Mittal A., Gupta D., Gupta S. (2015). Correlation Between Clinical Findings of Temporomandibular Disorders and MRI Characteristics of Disc Displacement. Open Dent. J..

[49] Boos-Lima F.B.D.J., Guastaldi F.P.S., Kaban L.B., Peacock Z.S. (2024). Accuracy of skeletal scintigraphy for the evaluation of mandibular growth disorders: a systematic review. Int. J. Oral Maxillofac. Surg..

[50] Huang S.C., Pareek A., Seyyedi S., Banerjee I., Lungren M.P. (2020). Fusion of medical imaging and electronic health records using deep learning: a systematic review and implementation guidelines. npj Digit. Med..

[51] Pi S.-W., Lee B.-D., Lee M.S., Lee H.J. (2023). Ensemble deep-learning networks for automated osteoarthritis grading in knee X-ray images. Sci. Rep..

[52] Ganaie M.A., Hu M., Malik A.K., Tanveer M., Suganthan P.N. (2022). Ensemble deep learning: A review. Eng. Appl. Artif. Intell..

[53] Mohammed A., Kora R. (2023). A comprehensive review on ensemble deep learning: Opportunities and challenges. J. King Saud Univ. Comput. Inf. Sci..

[54] Ali Y., Hussain F., Haque M.M. (2024). Advances, challenges, and future research needs in machine learning-based crash prediction models: A systematic review. Accid. Anal. Prev..

[55] Chisnoiu A.M., Picos A.M., Popa S., Chisnoiu P.D., Lascu L., Picos A., Chisnoiu R. (2015). Factors involved in the etiology of temporomandibular disorders - a literature review. Clujul Med..

[56] Smith S.B., Mir E., Bair E., Slade G.D., Dubner R., Fillingim R.B., Greenspan J.D., Ohrbach R., Knott C., Weir B. (2013). Genetic Variants Associated With Development of TMD and Its Intermediate Phenotypes: The Genetic Architecture of TMD in the OPPERA Prospective Cohort Study. J. Pain.

[57] Srikanth V.K., Fryer J.L., Zhai G., Winzenberg T.M., Hosmer D., Jones G. (2005). A meta-analysis of sex differences prevalence, incidence and severity of osteoarthritis. Osteoarthr. Cartil..

[58] Müller L., Kellenberger C.J., Cannizzaro E., Ettlin D., Schraner T., Bolt I.B., Peltomäki T., Saurenmann R.K. (2009). Early diagnosis of temporomandibular joint involvement in juvenile idiopathic arthritis: a pilot study comparing clinical examination and ultrasound to magnetic resonance imaging. Rheumatology.

[59] Altman R., Alarcón G., Appelrouth D., Bloch D., Borenstein D., Brandt K., Brown C., Cooke T.D., Daniel W., Gray R. (1990). The American College of Rheumatology criteria for the classification and reporting of osteoarthritis of the hand. Arthritis Rheum..

[60] Schiffman E., Ohrbach R., Truelove E., Look J., Anderson G., Goulet J.P., List T., Svensson P., Gonzalez Y., Lobbezoo F. (2014). Diagnostic Criteria for Temporomandibular Disorders (DC/TMD) for Clinical and Research Applications: recommendations of the International RDC/TMD Consortium Network∗ and Orofacial Pain Special Interest Group. J. Oral Facial Pain Headache.

[61] Raiaan M.A.K., Sakib S., Fahad N.M., Mamun A.A., Rahman M.A., Shatabda S., Mukta M.S.H. (2024). A systematic review of hyperparameter optimization techniques in Convolutional Neural Networks. Decis. Anal. J..

[62] Lee Y.-H., Jeon S., Auh Q.S., Chung E.-J. (2024). Automatic prediction of obstructive sleep apnea in patients with temporomandibular disorder based on multidata and machine learning. Sci. Rep..

[63] Metz C.E. (1978). Basic principles of ROC analysis. Semin. Nucl. Med..

