# Supplementary material for: Deep learning-based diagnosis of temporomandibular joint osteoarthritis using whole-body bone scans
Source: iScience. 2025 Nov 11;28(12):114027. doi: 10.1016/j.isci.2025.114027 (PMC12767182; doi:10.1016/j.isci.2025.114027)
Supplement: Document S1. Figures S1 and Tables S1 and S2 [file mmc1.pdf]

iScience, Volume *28*

Supplemental information

Deep learning-based diagnosis of temporomandibular joint osteoarthritis using whole-body bone scans

Yeon-Hee Lee, Hee-Sung Kim, Seonggwang Jeon, Q-Schick Auh, Il Ki Hong, Sunju Choi, Fernando Guastaldi, Hyungsoon Im, Yung-Kyun Noh, and Akhilanand Chaurasia

Supplementary Table 1. Sex-stratified AUC comparison across age groups with non-parametric permutation testing

Age group	n (Total)	n (Male)	n (Female)	Male AUC [95% CI]	Female AUC [95% CI]	p-value
10–19	147	52	95	0.7681 [0.490–1.000]	0.9162 [0.810–0.986]	0.206
20–39	334	106	228	0.8852 [0.813–0.943]	0.8653 [0.813–0.912]	0.656
40–59	242	36	206	0.9549 [0.876–1.000]	0.8782 [0.827–0.922]	0.188
Over 60	171	54	117	0.8090 [0.657–0.941]	0.9084 [0.846–0.960]	0.143

DeLong's test was used to evaluate statistical differences between male and female AUCs. AUC: Area Under the Curve; CI: Confidence Interval; n: sample size.

Supplementary Table 2. Pairwise permutation test for AUC differences between age subgroups (10–13, 14–16, 17–19, 20–39, 40–59, Over 60)

Age group 1	Age group 2	p-value (raw)	p-value (corrected, Holm-Bonferroni)
Over 60	14–16	0.0360	0.540
40–59	10–13	0.0500	0.700
40–59	14–16	0.0515	0.700
20–39	14–16	0.0570	0.700
Over 60	10–13	0.0995	1.000
20–39	10–13	0.1135	1.000
40–59	17–19	0.2245	1.000
17–19	14–16	0.2340	1.000
10–13	14–16	0.2410	1.000
20–39	40–59	0.4090	1.000
17–19	Over 60	0.4790	1.000
20–39	17–19	0.6385	1.000
40–59	Over 60	0.6555	1.000
17–19	10–13	0.7300	1.000
20–39	Over 60	0.8365	1.000

The results of pairwise permutation tests with Holm–Bonferroni correction were obtained to evaluate differences in model performance across age subgroups.
AUC: Area Under the Curve;
p-value (raw): uncorrected significance level from the permutation test;
p-value (corrected): significance level adjusted using the Holm–Bonferroni method.

Supplementary Figure 1. Bootstrap distributions of AUC estimates across age subgroups

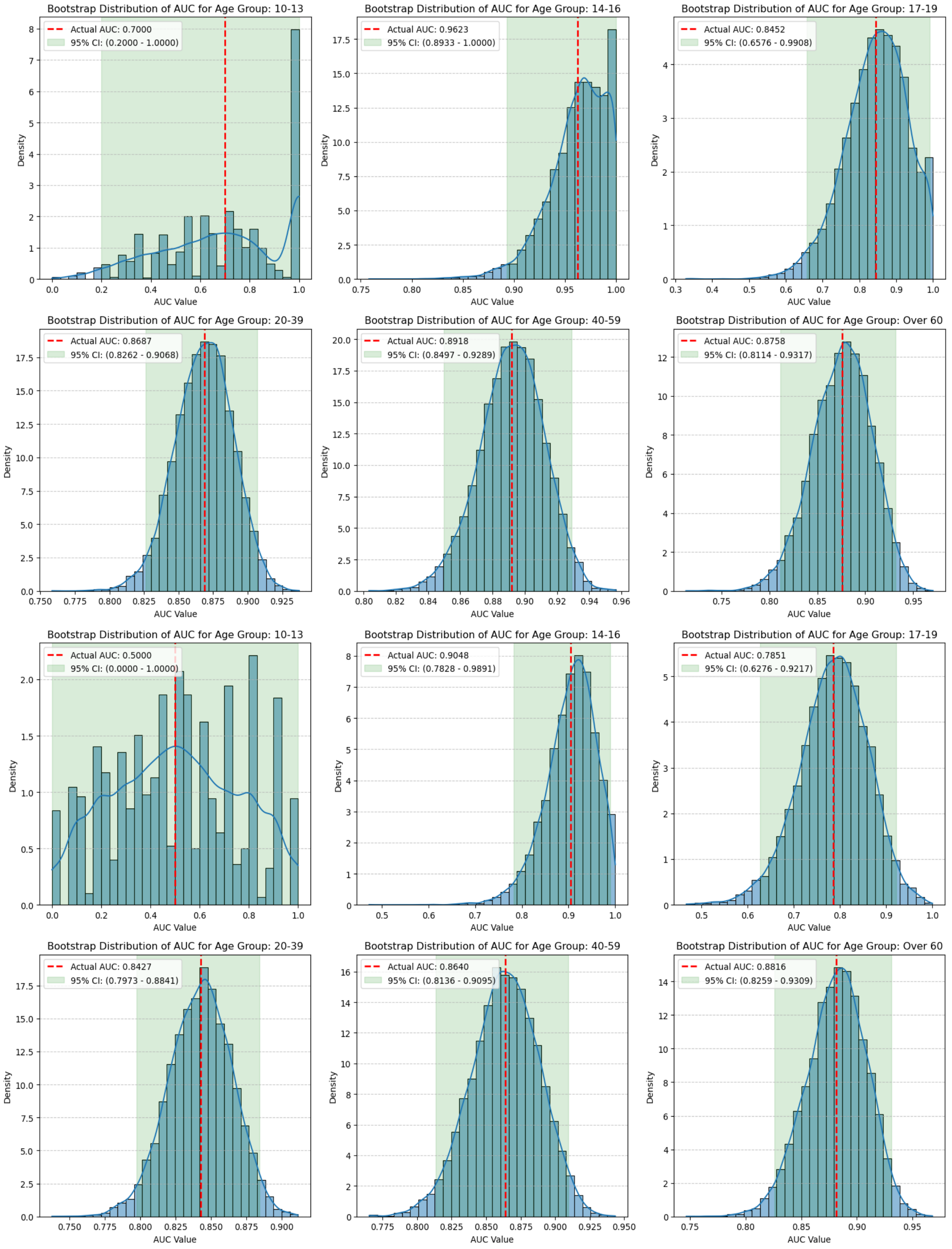

Bootstrap distributions of the AUC (Area Under the Curve) estimates for TMJ–OA prediction across different age subgroups (10–13, 14–16, 17–19, 20–39, 40–59, and Over 60 years). The vertical red dashed line indicates the actual AUC, and the green shaded region represents the 95% confidence interval of the bootstrap samples. In younger age groups—particularly 10–13 years—the bootstrap distributions are highly skewed and irregular due to the small sample size, violating the assumption of normality. These visual characteristics support the use of non-parametric statistical methods such as permutation testing rather than parametric approaches.
